# The Synergistic Mechanism of *Chelidonium majus* Alkaloids on Melanoma Treatment via a Multi-Strategy Insight

**DOI:** 10.3390/molecules29225412

**Published:** 2024-11-16

**Authors:** Peng Chen, Xin-Ye Ji, Jian-Ting Feng, Xiao-Qin Wang, Bo Zhang

**Affiliations:** 1 Key Laboratory of Medicinal and Edible Plants Resources Development of Sichuan Education Department, Sichuan Industrial Institute of Antibiotics, School of Pharmacy, Chengdu University, Chengdu 610106, China; liliflora_i@163.com (P.C.); wangxq5166@sina.com (X.-Q.W.); 2 Key Laboratory of Xinjiang Phytomedicine Resources and Utilization, Ministry of Education, School of Pharmacy, Shihezi University, Shihezi 832003, China; jxy_yx@163.com (X.-Y.J.); jianting2023@stu.shzu.edu.cn (J.-T.F.)

**Keywords:** melanoma, combination therapy, muti-target, network pharmacology

## Abstract

Melanoma represents a formidable challenge in dermatological oncology due to its resistance to conventional treatments. The Celandine Alkali Injection Formula (CAIF) offers benefits on clinical internal medicine treatments, within which chelidonine and tetrandrine are recognized as potential quality markers. However, their synergistic mechanisms facilitating their anti-melanoma action remain unveiled. This study embarked on an exploration of CAIF’s therapeutic potential through a multifaceted research design, integrating system pharmacological predictions with empirical molecular biological evaluations. The dual application of chelidonine and tetrandrine within CAIF exhibited a pronounced inhibitory effect on the proliferation of B16F10 cells, surpassing the effectiveness of individual compound administration. Computational predictions identified the top 50 targets, involved in key signaling pathways including cell cycle regulation, and melanogenesis. RNA sequencing further elucidated that the combinatory treatment modulated a broader spectrum of differentially expressed genes, implicating crucial biological processes including cell differentiation, and tyrosinase metabolism. The combination markedly enhanced melanogenesis and apoptotic indices, arrested cell cycle progression, and fostered cellular differentiation. Notably, chelidonine additionally curtailed the migratory capacity of B16F10 cells. Our findings underscore the therapeutic potential of chelidonine and tetrandrine, key components of CAIF, in effectively combating melanoma by targeting cell proliferation, migration, differentiation, and melanogenesis.

## 1. Introduction

Melanoma, the most lethal form of skin cancer, is characterized by the rapid proliferation of melanocytes, the cells responsible for pigment production in the skin [1]. Its aggressive nature, underscored by a high propensity for recurrence and metastasis, makes it a formidable challenge in clinical oncology. The complexity of melanoma treatment is exacerbated by its resistance to conventional therapies such as chemotherapy and immunotherapy, necessitating the exploration of novel therapeutic strategies [2]. However, these treatments often come with serious side effects and the risk of drug resistance [3].

At the molecular level, melanoma is driven by genetic mutations in key regulatory genes, including BRAF, NRAS, and c-KIT, which are implicated in cell growth, differentiation, and survival pathways. The discovery of these mutations has led to the development of targeted therapies [4,5]. However, the emergence of resistance and the occurrence of severe side effects limit their long-term efficacy. Thus, there is an urgent need for innovative approaches that can circumvent these issues.

Recent research has highlighted the potential of differentiation therapy in treating melanoma. This strategy aims to induce malignant cells to revert to a less aggressive, more differentiated state, potentially reducing their proliferative and metastatic capabilities [6]. The concept of differentiation therapy is not new and has been successfully applied in the treatment of acute promyelocytic leukemia with retinoic acid and arsenic trioxide, showcasing the potential for reprogramming cancer cell behavior [7,8].

In this evolving therapeutic landscape, chelidonine, from *Chelidonium majus* L. (*C. majus*), and tetrandrine, from *Stephania tetrandra* S. Moore, have garnered attention for their antitumor activities [9,10]. These compounds have been shown to interact with multiple cellular targets and signaling pathways involved in cancer progression, including those related to apoptosis, cell cycle regulation, and angiogenesis [11,12]. Preliminary studies suggest that these alkaloids can inhibit tumor growth and induce cell differentiation in various cancer models, making them promising candidates for melanoma treatment.

Moreover, the use of chelidonine and tetrandrine in combination, as seen in traditional formulations like the Celandine Alkali Injection Formula, poses an intriguing possibility [13]. This formula, known for its analgesic and antitumor effects, exemplifies the potential of combining natural compounds to enhance therapeutic outcomes. The synergistic effects observed in preliminary studies point towards a multifaceted mechanism of action, potentially overcoming the limitations of single-agent therapies.

**Figure 1:**
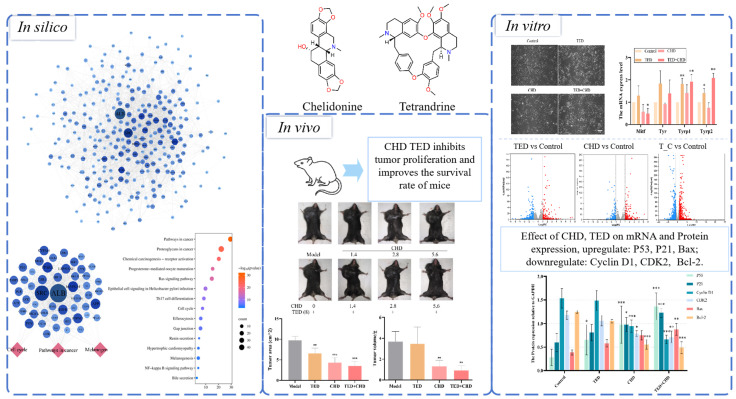
Flowchart of present study *in silico*, *in vivo*, and *in vitro*. * *p* < 0.05 vs. control group, ** *p* < 0.01 vs. control group, *** *p* < 0.001 vs. control group.

Building upon this foundation, our study aims to rigorously evaluate the antitumor efficacy of chelidonine and tetrandrine, both independently and in synergy, against melanoma. By employing comprehensive *in vivo* and *in vitro* experimental models, this research seeks to elucidate the mechanisms through which these alkaloids affect tumor proliferation, melanin production, cell cycle dynamics, and differentiation (Figure 1). Such insights are crucial for advancing the development of differentiation therapy as a novel and effective approach to melanoma treatment, offering hope for improved patient outcomes in the face of this challenging disease.

## 2. Results

### 2.1. Chelidonine and Tetrandrine as Active Anti-Tumor Components in Celandine Alkali Injection Formula

The main alkaloid components of the Celandine Alkali Injection Formula (CAIF) were exacted from *Chelidonium majus* L., *Stephania tetrandra* S. Moore, and *Corydalis yanhusuo*. Using the keyword “melanoma” as the screening condition in the Herb database and the SymMap database, it was found that *Chelidonium majus* L. and *Stephania tetrandra* S. Moore were correlated with a variety of melanoma species compared with *Corydalis yanhusuo* (Table S1).

To ascertain the bioactive components responsible for exerting anti-melanoma activity in the two herbs in a systematic view, a thorough search was conducted using the Herb and TCMSP databases, specifically targeting *Chelidonium majus* L. and *Stephania tetrandra* S. Moore. The search revealed that a total of 27 out of 37 compounds and 12 out of 50 alkaloids were commonly present in both herbs, as summarized in Table S2. A hierarchical clustering analysis was further employed to analyze the identified compounds, revealing that alkaloids constituted a significant proportion of the bioactive components (Figure S1). By incorporating the analysis of characteristic functional groups associated with alkaloids, the percentage of alkaloids within the herbal extracts was determined to be 37 out of 77. This finding aligns with previous reports indicating alkaloids as the primary bioactive components in the CAIF.

**Figure 2:**
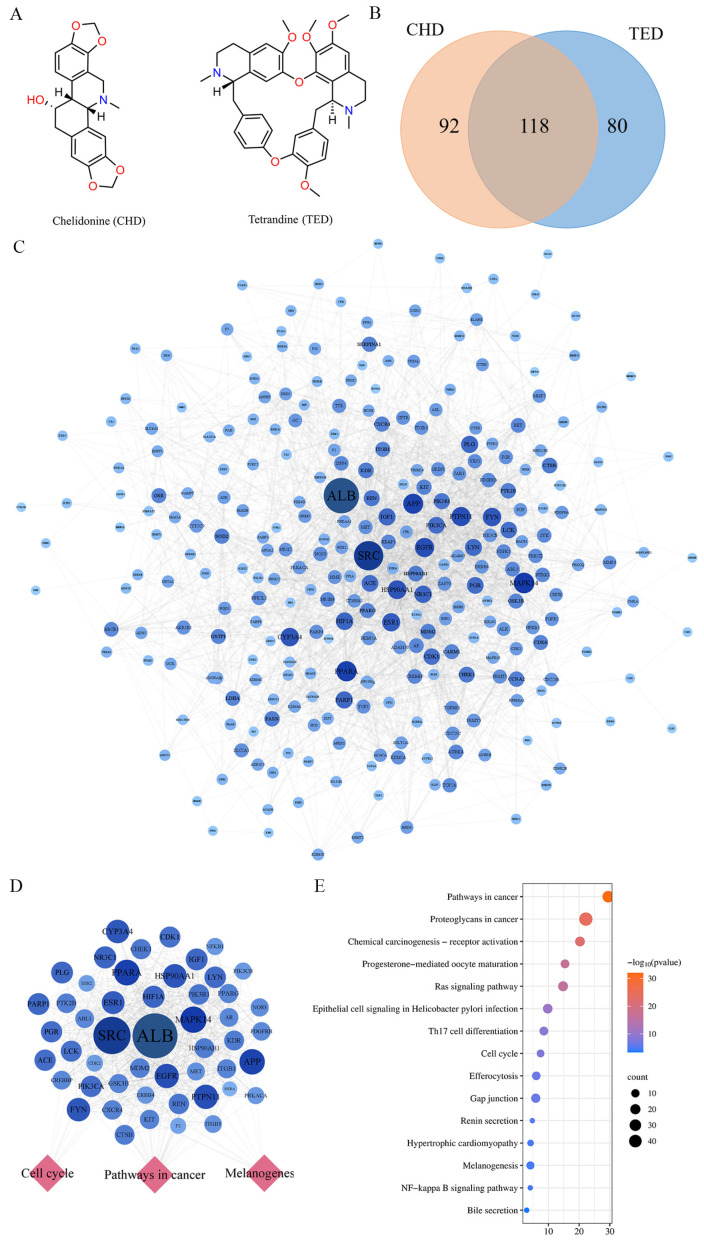
The number of potential targets for chelidonine and tetrandrine. (**A**) shows the structures of chelidonine and tetrandrine. (**B**) shows the number of targets predicted by CHD and TED, where the numbers inside the parentheses are the number of genes eligible for screening. (**C**) shows the potential targets of TED and CHD action. The circle represents the target protein. The darker the color, the larger the diameter of the circle, which represents a greater degree value. (**D**) indicates in the top 50 targets of the PPI network and targets–pathways network. (**E**) Top 50 targets of KEGG pathway enrichment analysis.

Chelidonine (CHD) is the active ingredient in *Chelidonium majus* L., and it is a quality marking ingredient in another *Chelidonium majus* injection [14]. In the Chinese Pharmacopoeia, tetrandrine (TED) is one of the quality markers of *Stephania tetrandra* S. Moore [15]. By observing the structures of CHD and TED, it was found that they both have some symmetry and fewer hydrophilic groups (Figure 2A). To better explain their mechanisms, CHD and TED were predicted using a computer network prediction platform, and 290 targets were collected through the PharmMapper database and the SuperPred database, among which 92 and 80 targets were CHD and TED, respectively, and 118 common targets (Figure 2B).

The 290 targets were imported into the STRING database to construct a PPI network (Figure 2C). The PPI network was uploaded to Cytoscape, for a total of 280 nodes and 2588 edges. The top 50 targets ranked by degree in cytoHubba application are shown in Table S3. The 50 targets abovementioned were imported into the metascape database for pathway enrichment analysis, and the results are shown by selecting the term content with the *p*-value < 0.01; 146 KEGG pathways were obtained (Table S4). The top 50 targets were mainly involved in KEGG signaling pathways such as pathways in cancer, cell cycle, and melanogenesis (Figure 2D,E). The results above show that CHD and TED, as important components, were highly correlated with antitumor in the CAIF.

### 2.2. Chelidonine and Tetrandrine Inhibited the Growth of Melanoma In Vivo and In Vitro

**Figure 3:**
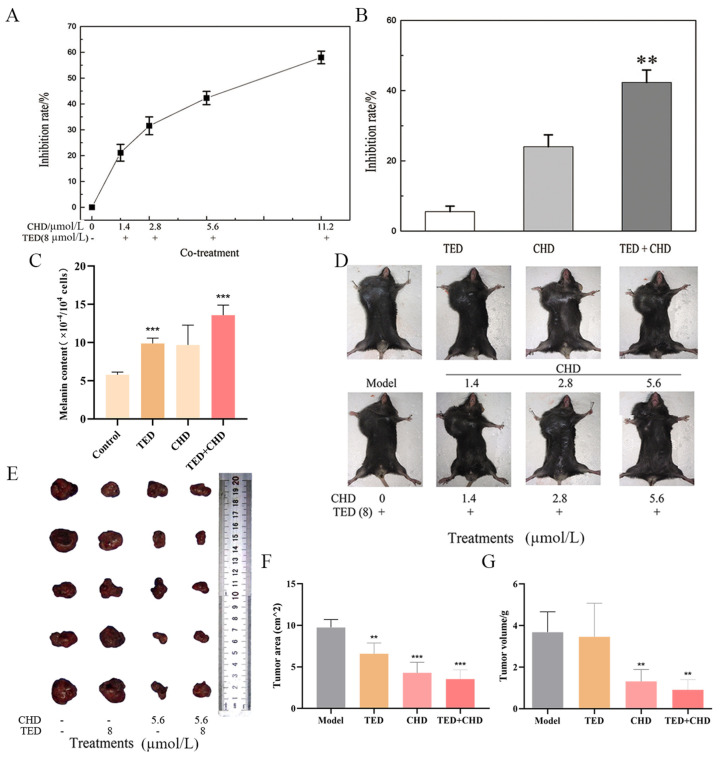
Chelidonine and tetrandrine inhibited B16F10 cells and tumor growth. (**A**) shows that cells were treated with different concentrations of CHD and TED. Fixed 8 μmol/L of TED and different concentrations of CHD (0, 1.4, 2.8, 5.6, 11.2 μmol/L) on B16F10 cells for 24 h. (**B**) shows 8 μmol/L TED, and 5.6 μmol/L on B16F10 cells. (**C**) shows the melanin content on the B16F10 cell was detected. (**D**) shows the treatment in the mice’s body after inoculating the tumor. Experiments were divided into three groups: the model group, the individually administered group with different concentrations of CHD, and the co-treatment group. (**E**–**G**) show that the tumor was removed from the mice’s bodies with varying doses of the two compounds, and tumor volume and size were measured. ** *p* < 0.01 vs. control group, *** *p* < 0.001 vs. control group.

To investigate the antitumor activity of chelidonine (CHD) and tetrandrine (TED) on melanoma cells, an MTT assay was conducted to assess their impact on the growth of B16F10 melanoma cells. Our results demonstrated that CHD, administered at various concentrations, along with a fixed concentration of TED (8 μmol/L), effectively suppressed B16F10 cell proliferation. This inhibitory effect was dose-dependent, with higher concentrations of CHD exhibiting stronger antiproliferative activity (Figure 3A). Notably, while TED alone at 8 μmol/L did not significantly affect cell proliferation, its combination with CHD resulted in a significant increase in the inhibition rate. Specifically, at a concentration of 5.6 μmol/L, CHD alone inhibited B16F10 cell proliferation by 26.0%, while the combined treatment with TED increased the inhibition rate to 57.7%, exceeding the cumulative effect of the individual drugs (Figure 3B).

To detect the effect of CHD and TED on the tumor growth of melanoma *in vivo*, a melanoma model was established in C57BL/6 mice; to validate the drug efficacy, various concentrations of CHD were combined with a consistent concentration of TED, and the two compounds were mixed in differing proportions to assess their impact on reducing tumor volume in the treatment group relative to the model group. As shown in Figure 3D–G, the tumor growth inhibition effects of 8 μmol/L TED, and 1.4, 2.8 and 5.6 μmol/L CHD were not obvious, but the tumor inhibition effect of the combination was significantly better than that of the single-agent group, and the tumor inhibition effect of the combination with 5.6 μmol/L CHD and 8 μmol/L TED was the best.

### 2.3. Identification of Gene Differential Expression and KEGG Enrichment Analysis

**Figure 4:**
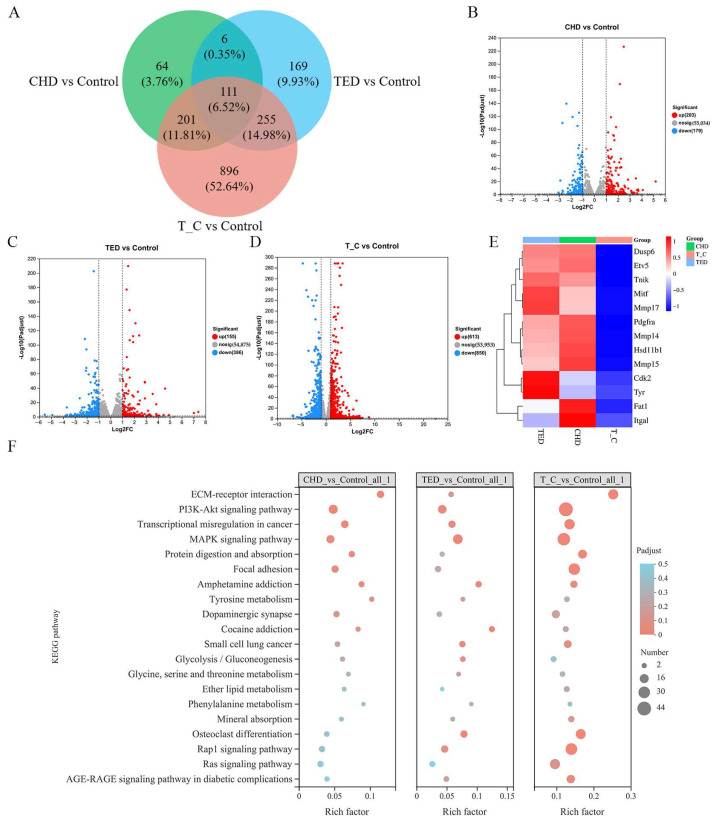
RNA-seq analysis of CHD, TED, and co-treatment. (**A**) Venn diagram of differential genes in three experimental groups in RNA-seq. (**B**–**D**) RNA-seq gene volcano diagram in three experimental groups. (**E**) Melanoma-related gene heat map in three experimental groups. (**F**) KEGG enrichment analysis of differential genes in three experimental groups.

To elucidate the therapeutic disparities among CHD, TED, and the co-treatment group in B16F10 cells, we conducted a comprehensive analysis of gene expression alterations utilizing RNA-Seq data, comparing them with the control group. The analysis revealed a total of 2386 genes exhibiting significant modifications in expression levels, encompassing both upregulation and downregulation. The volcano plot effectively highlighted the total count of differentially expressed genes influenced by CHD and TED. Specifically, TED modulated 541 genes, with 155 genes upregulated and 386 genes downregulated. In contrast, CHD regulated 382 genes, comprising 203 upregulated genes and 179 downregulated genes. Notably, the co-treatment group exhibited the highest regulatory activity, modulating 1463 genes, including 613 upregulated genes and 850 downregulated genes. This observation suggests a more pronounced transcriptional response in the co-treatment group compared to the TED and CHD groups (Figure 4A–D). Furthermore, among the differentially expressed genes associated with melanoma, the combination group significantly suppressed the transcript levels of key genes such as mitf, cdk2, and tyr (Figure 4E), highlighting its potential therapeutic efficacy.

Furthermore, KEGG enrichment analysis of genes with altered expression levels was performed. It significantly affects many signaling pathways, such as the extracellular matrix (ECM) interactions, MAPK signaling pathway, focal adhesion signaling pathway, and tyrosine metabolism signaling pathway, which were highly selective for melanoma multi-phenotypes (Figure 4F). The analysis of the RNA-seq results showed that the therapeutic effect of the co-treatment was stronger than that of the TED group or the CHD group.

### 2.4. Chelidonine Decreased the Migration Ability of B16F10 Cells

**Figure 5:**
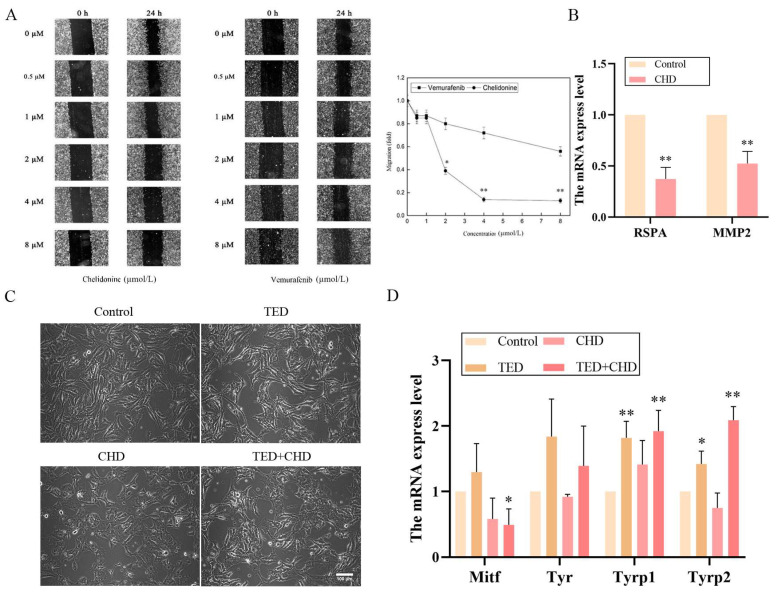
Inhibitory roles of cell migration and cell differentiation in B16F10 cells. (**A**) shows the effect of concentrations (0, 0.5, 1, 2, 4, and 8 μmol/L) of CHD and vemurafenib after 0 h and 24 h, wounding-healing assay, and quantification of the migration distance of B16F10 cells. (**B**) shows the PCR array results about cell migration genes in CHD. (**C**) shows morphology changes in B16F10 cells treated with TED, CHD, and co-treatment for 24 h. (**D**) qRT-PCR shows the *mitf*, *tyr*, *tyrp1*, and *tyrp2* mRNA expression. * *p* < 0.05 vs. control group, ** *p* < 0.01 vs. control group.

Cell migration is an important process involved in cancer metastasis. Differential gene GO enrichment analyses in biological processes showed that CHD was correlated with cell migration and cell motility processes compared to TED; also, CHD and TED regulate cell differentiation (Figure S2). Therefore, we chose CHD for cell migration experiments. To investigate the effects of chelidonine on B16F10 cell migration ability, a wound-healing test was performed [16]. Various chelidonine concentrations (0.5, 1, 2, 4, and 8 μmol/L) were used. Vemurafenib, a clinical medicine used to treat melanoma, was added with the same concentrations to culture media to assess its influence on cell migration. The addition of 8 μmol/L CHD decreased cell migration to 90% of that of the control (Figure 5A). Compared to the control group, vemurafenib significantly reduced cell migration rate after 24 h of culture at the same concentration, with no significant difference observed (Figure 5A). In addition, the PCR array results show that CHD regulates multiple targets related to cell migration (Figure S3). To understand the mechanisms of chelidonine on B16F10 cells, a PCR array assay was applied to investigate the effects on migration gene expression, such as that of *mmp2*, *mmp9*, and *rspa*. As shown in Figure 5B, CHD significantly downregulated the mRNA expression of *mmp2* and *rspa*.

In the observation of cell morphology, compared with the control group, CHD and TED had little effect on cell differentiation (Figure 5C). TED showed little change in cell morphology, but after the CHD and co-treatment group, the cytoplasm of B16F10 cells became larger and cell branching was reduced. qRT-PCR was adopted to examine the expression of the related melanogenesis and cell differentiation gene, and, as indicated in Figure 5D, the relative mRNA levels of *mitf* after co-treatment were significantly downregulated, while those of *tyrp1* and *tyrp2* were significantly upregulated (*p* < 0.05).

### 2.5. The Effects of Chelidonine and Tetrandrine on Cell Apoptosis and Cell Cycle

**Figure 6:**
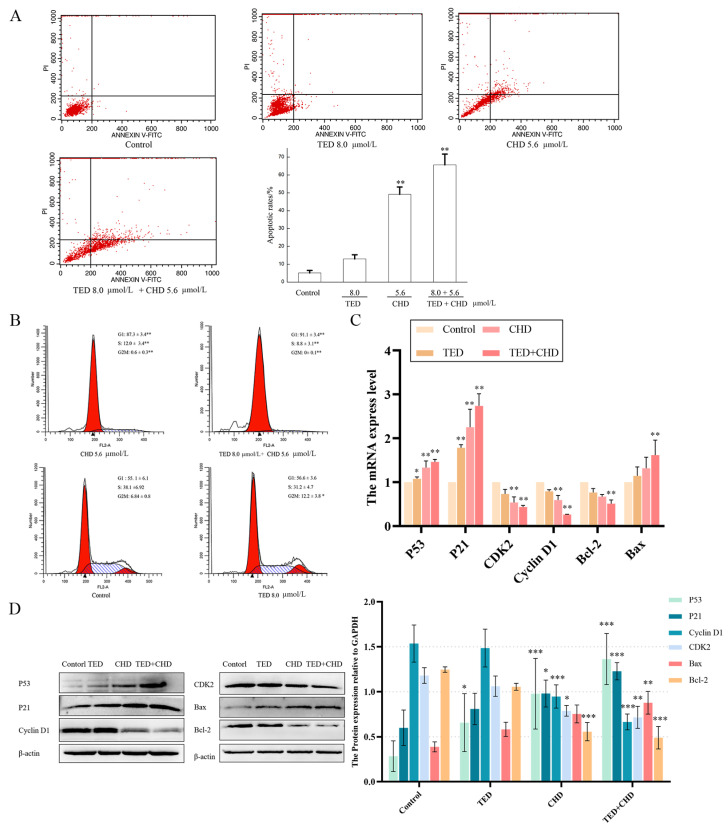
Apoptosis induction and cell cycle arrest on B16F10 cells. (**A**) Annexin V-PI-staining analysis was performed to evaluate apoptotic cell death in B16F10 cells after CHD, TED, and co-treatment. (**B**) PI-staining analysis was performed to evaluate the cell cycle change in B16F10 cells after CHD, TED, and co-treatment. (**C**) qPCR products showed the effects of CHE, TED, and co-treatment on mRNA expression of *p53*, *p21*, *cyclin D1*, *cdk2*, *bax*, and *bcl-2* against *gapdh*. (**D**) Western blot analysis of indicated proteins in CHD, TED, and co-treatment group. * *p* < 0.05 vs. control group, ** *p* < 0.01 vs. control group, *** *p* < 0.001 vs. control group.

To test the apoptosis and cell cycle inductivity of CHD and TED on B16F10 melanoma cells, we found that cells treated for 24 h with 8 μmol/L of TED revealed the rate of early apoptosis was 12.9%, the apoptosis rate of CHD with 5.6 μmol/L treatment was 49.1%, and both early and late apoptosis were observed. The apoptosis rate increased to 65.6% in the co-treatment group, and the number of late-apoptosis cells increased significantly (Figure 6A).

PI staining analysis was performed to evaluate the change in cell cycle in B16F10 cells. In B16F10 cells treated after 24 h with 8 μmol/L of TED and 5.6 μmol/L of CHD, respectively, the percentage of cells detected in the G1 phase was (55.6 ± 3.6)% and (87.3 ± 3.4)%, and the proportion of cells in G2/M phase was (12.2 ± 3.8)% and (10.6 ± 0.3)%. The cycle arrest of TED was not obvious, and CHD may cause significant G1 cell arrest and lead to the absence of cells in the G2/M phase (Figure 6B). The proportion of G1 cells in the co-treatment group was as high as (91.1 ± 3.4)%, mainly to reduce the S phase cells, from which we can see that the combination of drugs caused a significant retardation of the G1 phase, and there was a significant difference compared with the control group (** *p* < 0.01). To further examine the observed changes in apoptosis and cell cycle genes on B16F10 cells, the mRNA and protein levels of *p53*, *p21*, *cyclin D1*, *cdk2*, *bax*, and *bcl*−*2* were determined. The mRNA and protein expression of p53, p21, and Bax were significantly increased compared with the control group, and those of Cyclin D1, CDK2, and Bcl−2 were significantly decreased compared with the control group (Figure 6C, D). The outcomes revealed induced apoptosis and inhibited cell cycle of the treatment group on B16F10 cells by CHD and co-treatment.

## 3. Discussion

The landscape of melanoma treatment has undergone significant evolution in recent years, marked by a transition from traditional cytotoxic chemotherapies to more sophisticated approaches such as targeted therapies, immunotherapy, and combinational strategies. Dacarbazine, the only FDA-approved cytotoxic agent for melanoma, primarily functions by disrupting DNA synthesis, thus inhibiting tumor growth, albeit as a second-line treatment due to its side effects [17]. The advent of targeted therapies was propelled by the high mutagenicity characteristic of melanoma, with mutations in genes like BRAF NRAS, and P53 playing pivotal roles in its pathogenesis [2]. However, the utility of these agents is often marred by the onset of toxic reactions in patients.

Immunotherapeutic agents, including anti-PD1 and anti-CTLA−4 antibodies, have shown promise with relatively lower side effects, and their efficacy can be enhanced when used in combination with other drugs [18]. In particular, the combination of dabrafenib and trametinib has gained traction in Asia, supplemented with adjuvant therapies to manage side effects [19]. Traditional Chinese medicine, with its use of herbal compounds like Ginkgo biloba polysaccharides, offers an integrative approach by enhancing efficacy and mitigating toxicity when combined with Western medicine [20]. Notably, fangchinoline, a herbal compound, has demonstrated the ability to induce apoptosis in melanoma cells, particularly when used alongside conventional chemotherapeutics like gemcitabine [21].

The potential of natural products, especially alkaloids such as chelidonine and tetrandrine, in anti-melanoma multi-strategies is underscored by their ability to induce apoptosis in melanoma cells. The observed synergistic effect of chelidonine and tetrandrine in melanoma treatment aligns with the growing body of evidence supporting combination therapy as a cornerstone in cancer treatment strategies. The notion that combining agents can enhance therapeutic efficacy while potentially reducing side effects is particularly relevant in melanoma given its heterogeneity and resilience to monotherapies. B16F10 cells are a melanoma cell line derived from C57BL/6 mice. They have a high metastatic ability and are often used to study the metastatic mechanism of melanoma and the screening of antitumor drugs [22]. Previous studies have shown the efficacy of chelidonine in reducing tumor size in melanoma-afflicted C57BL/6J mice and augmenting the antitumor effects of lenvatinib [23]. Tetrandrine, on the other hand, has been noted for its role as a chemosensitizer and toxicity mitigator when used in conjunction with other chemotherapeutic agents [24]. The pharmacological results indicate that CHD had a good effect of inhibiting the growth of melanoma, which was significantly enhanced when combined with TED. It was suggested that the combination of these two alkaloids not only targets melanoma cells more effectively but might also mitigate resistance mechanisms that typically emerge with single-agent treatments.

Recent studies have provided insights into the molecular mechanisms underlying the effectiveness of such combinations. For instance, research into the role of autophagy in cancer therapy has shown that manipulating this process can significantly affect the sensitivity of melanoma cells to chemotherapeutic agents [25]. Autophagy, a cellular degradation process, can act both as a tumor suppressor and a factor in tumor cell survival depending on the context and stage of cancer development [26]. Chelidonine has been demonstrated to induce autophagy in certain types of cancer cells, which could complement its apoptotic effects [27]. Tetrandrine, meanwhile, is known to modulate calcium signaling within cells, a pathway intimately connected with both autophagy and apoptosis [28]. The interplay between these processes may contribute to the observed increased efficacy of the combination treatment, suggesting a multi-target approach to inducing melanoma cell death.

Our investigation reveals the multifaceted nature of melanoma therapy, where the aim is not solely to target the lethality of tumor cells but to induce differentiation and halt their proliferative capabilities. Chelidonine and tetrandrine have been found to interact with several melanoma-related KEGG pathways, influencing cell cycle dynamics, differentiation, and melanogenesis. These findings are consistent with RNA-seq analyses and experimental studies which show that these compounds, individually or in combination, affect the expression of key genes like *mitf*, *tyr*, and *tyrp1/2*, which are crucial for melanin synthesis and cellular differentiation [6,29].

Furthermore, our research highlights the importance of cell cycle regulation, apoptosis, and migration in melanoma progression. The combination of chelidonine and tetrandrine was observed to arrest cells in the G1 phase, downregulate oncogenic markers like cyclin D1, Cdk2, and Bcl-2, and promote apoptosis. Additionally, these compounds significantly impacted cell differentiation and migration, as evidenced by RNA-seq GO enrichment analysis and cell wound healing assays. The downregulation of *mmp2* and *rpsa*, markers associated with melanoma invasion, further substantiates the role of these alkaloids in inhibiting melanoma progression [30,31]. Moreover, the downregulation of *mmp2* and *rpsa* by chelidonine highlights another crucial aspect of melanoma pathology—the degradation of the extracellular matrix (ECM), which is a key step in tumor invasion and metastasis. MMP2, a matrix metalloproteinase, is directly involved in the breakdown of collagen and gelatin components of the ECM, facilitating cancer cell migration [32]. The inhibition of *mmp2* and *rpsa* not only suggests a mechanism through which the compounds prevent the spread of melanoma cells but also aligns with the broader need for therapies that target the invasive nature of this cancer.

Chelinonine is an isoquinoline alkaloid that has been evaluated by many authors as a potent anti-proliferative and proapoptotic agent against various types of cancer cells, including melanoma cells [33]. Advanced melanomas are characterized by multiple pathogenic mutations. Recent studies indicate that chelidonine treatment affects genes including the key signaling pathways that govern proliferation (BRAF, NRAS), and resistance to apoptosis (TP53) [34,35,36,37]. STK19 kinase was a potential therapeutic target for NRAS-mutant melanomas; 5 and 20 µmol/L chelidonine inhibited STK19, and reduced the phosphorylation of NRAS S89, MEK, ERK1/2, and AKT in all four NRAS-mutant melanoma cell lines [36]. Chelidonine exhibited strong anti-proliferative activity in A−375 and SK-MEL−2 cells with wild-type p53 [34]. Furthermore, chelidonine predominantly induced apoptosis in OCM−1 cells, and exerted opposing effects on IL−6-induced activation and constitutive serine phosphorylation of STAT3 in OCM−1 and OCM−3 human primary uveal melanoma cells [33,38]. Due to the differences in membrane receptors between normal and tumor cells, the ability of drugs to enter cells varies, and monitoring the decrease in alkaloid concentration in the cell medium revealed that chelidonine at low concentrations and with short exposure times exhibits significant penetration into B16F10 melanoma cells [35]. Nonetheless, the specific mechanism behind this remains unclear. Additionally, although isoquinoline has potential hepatotoxicity, 20 mg/kg chelidonine had no significant apparent effects on hepatic function in C57BL/6 mice [36,39,40]. Therefore, it is evident that chelidonine possesses the capability to suppress the growth of certain melanoma cells, yet it is not without its limitations in terms of efficacy.

Isoquinoline alkaloids constitute the most abundant and significant active compounds in *Chelidonium majus* L., offering a substantial material foundation for its pharmacological actions [41]. Both benzylisoquinoline alkaloids (Benzophenanthridines, protoberberines, and protopines) and isoquinoline alkaloids (Aporphines) are considered to be the bioactive ingredients of *C. majus* and exhibit considerable pharmacological activity [35,42,43]. Isoquinoline alkaloids exhibit potent antiproliferative effects, and induce differentiation on melanoma cells [44,45]; sanguinarine, chelerythrine, chelilutine, and sanguilutine have also shown the ability to inhibit the proliferation of melanoma cells [33,34,35]. An increasing number of these alkaloids, which are structurally diverse due to their nitrogen atoms, have been reported to possess a wide range of pharmacological activities, including significant analgesic, anti-inflammatory, and antitumor effects [34,45]. Also, tetrandrine is a bisbenzylisoquinoline alkaloid [10], and our study demonstrated both pro-apoptotic and anti-migratory effects against B16F10 melanoma cells, and these activities were synergistically enhanced by the addition of tetrandrine. Therefore, given the structural uniqueness and similarity of *Chelidonium majus* alkaloids, these hold significant research potential to enhance antitumor efficacy by either modifying these compounds or employing combinations of them in the future.

In light of these findings, it is imperative to consider the broader implications for melanoma treatment paradigms. The integration of natural products such as chelidonine and tetrandrine into melanoma therapy could represent a shift towards more holistic and personalized treatment approaches. Such strategies would not only aim to eradicate tumor cells but also modulate the tumor microenvironment, enhance cellular differentiation, and reduce the likelihood of metastasis [44,46]. Our study suggests a potential induction mechanism of melanin augmentation and cell differentiation in the treatment groups, with the combined treatment of CHD and TED producing a more pronounced effect. Future research should focus on elucidating the precise molecular interactions between these compounds and melanoma pathways, optimizing combination dosages, and conducting comprehensive clinical trials to evaluate efficacy and safety in a broader patient population [47]. Additionally, the role of natural products in cancer treatment underscores the importance of biodiversity and the need for conservation efforts to preserve natural habitats that are potential sources of novel therapeutics [48]. The exploration of natural compounds in cancer research is a promising frontier, with the potential to yield treatments that are both effective and have minimal side effects compared to conventional chemotherapeutics.

**Figure 7:**
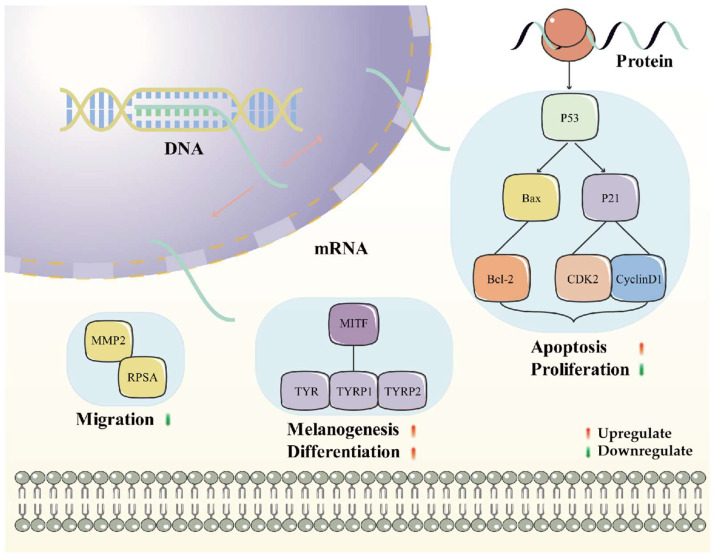
The underlying mechanism of chelidonine and tetrandrine.

By integrating the abovementioned results, we summarized that the combination of multiple targets may be the underlying mechanism of chelidonine and tetrandrine (Figure 7) for melanoma. It mainly includes the suppression of the cell cycle signaling pathway by inhibiting CDK2 and Cyclin D1, promoting P53 and p21, and the modulation of cellular MITF, TYR, TYRP1, and TYRP2 for melanogenesis and differentiation, and the ability to inhibit cell migration (MMP2 and RPSA) by chelidonine. This also showed us that chelidonine and tetrandrine inhibited melanoma through a multi-target strategy, providing a new perspective for drug combinations.

## 4. Materials and Methods

### 4.1. Materials

Chelidonine was purchased from Shanghai Yuanye Biotechnology Co., Ltd. (Purity 98%, Shanghai, China). Tetrandrine was bought from Sigma Chemical Co. (Purity 98%, Hong Kong, China). Fetal bovine serum (FBS) was attained from Sijiqing (Hangzhou Sijiqing Co., Hangzhou, China), and methyl thiazolyl tetrazolium (MTT) and dimethyl sulfoxide (DMSO) were purchased from Sigma Chemical Co. (St. Louis, MO, USA). Gentamicin was obtained from Shandong Sunrise Pharmaceutical Co., Ltd. (Zibo, China). All other chemicals were of analytical grade and were commercially available.

### 4.2. Target Prediction and Protein–Protein Interaction Network Construction

The structural formulas of chelidonine and tetrandrine were selected according to the PubChem database [49]; further corresponding targets were confirmed by the PharmMapper database and the SuperPred database [50,51]. A screening of potentially useful targets (PharmMapper: Norm fit ≥ Average; SuperPred: Probability ≥ 50%) was carried out for subsequent analysis. All collected targets were imported into the UniProt database and converted into official protein names. The targets were converted to the STRING for constructing target interaction network [52], enrichment was analyzed by the Metascape database [53], and the final network was generated by Cytoscape 3.9.1 [54].

### 4.3. Cells and Animal Treatments

The melanoma cell line B16F10 was purchased from the Cancer Cell Repository of Shanghai Cell Bank, Shanghai, China. B16F10 cells were cultured at 37 °C in 5% CO_2_, and the medium maintained in DMEM, supplemented with 10% FBS, and 1% penicillin/streptomycin (P/S). The cytotoxic activity of chelidonine, tetrandrine, and co-treatment were evaluated using the MTT assay. B16F10 cells were harvested during the growth of the exponential phase and plated in 96-well plates. Chelidonine was prepared in DMSO and diluted subsequently with the medium before use. B16F10 cells were exposed to chelidonine for 24 h, and then 10 μL of 5 mg/mL of MTT was added to each well for 4 h [55]. The reaction was halted by the addition of 150 μL DMSO, and the absorbance (A) at 570 nm was determined by spectrophotometry (Varioskan Flash 3001, Thermo, Waltham, MA, USA). The inhibition rate of the cell was calculated as follows:

Inhibition rate (%) = [(control group A values − experimental group A values)/control group A values] × 100%

The same approach was used for tetrandrine and the co-treatment.

All experimental protocols were approved by the Animal Investigation Committee at Shihezi University and adhered to the Ethical Guidelines of the International Association for the study of tumor (No. A2024-009-01). Then, 1 × 10^7^ mL B16F10 cells were suspended in PBS and inoculated into the right armpit of the mice; each mouse was injected with 0.2 mL cells, and the mice were sacrificed 10 days after the injection of tumor cells [36]. The experiment was divided into four groups: the model group, the different doses of chelidonine group, the same amount of tetrandrine group, and the co-treatment group. The animals were stratified so that the mean tumor sizes in all treatment groups were nearly identical. In order to minimize the potential suffering of the animals, all possible measures were taken.

### 4.4. RNA-Seq and PCR Array Data Analysis

Total RNAs were prepared with Trizol Reagent, and all operations were performed in a sterile environment. Sequencing of total RNA from B16F10 cells treated with TED, CHD, or TED + CHD was performed in Majorbio Bio-pharm Technology Co., Ltd. (Shanghai, China) using Illumina novaSeq 6000 (San Diego, CA, USA). The data were analyzed on the online platform of Majorbio Cloud Platform (www.majorbio.com) [56]. The significance of the data was satisfied by *p*-adjust < 0.05 and 2-fold change.

Changes in the expression of metastasis-related genes were detected using the RT2 Profiler Mouse Tumor Metastasis PCR Array (PAMM-028ZR; Qiagen, Hilden, Germany). cDNA was synthesized using RT2 First Strand Kit (Qiagen). RT2 SYBR Green qPCR Master Mix was used for the reaction following the manufacturer’s instructions. Amplification and real-time analysis were performed with Rotor-Gene Q 100 (Qiagen). The fold change was calculated by determining the ratio of mRNA levels to the control values using the Δ threshold cycle (Ct) method (2^−ΔΔCt^). All data were normalized to an average of five housekeeping genes: Gusb, Hprt, Hsp90ab1, Gapdh, and β-actin. PCR conditions used: hold for 10 min at 95 °C, followed by 40 cycles of 15 s at 95 °C and 30 s at 60 °C.

### 4.5. Wound-Healing Assay

The effect of chelidonine and vemurafenib on B16F10 cell migration was investigated as follows [57]: B16F10 cells were plated in 6-well plates and incubated at 37 °C in 5% CO_2_ until covered at the bottom of the plate. B16F10 cells were scraped off with a sterile pipette tip and rinsed with phosphate-buffered saline (PBS) twice. DMEM without FBS as the control and different concentrations of chelidonine and vemurafenib were added. The scratches were photographed when the scratch (0 h) was made and 24 h later. The width of the denuded area was measured using an electronic grid, and the distances crossed by the cells were determined. The mean of the controls was set to 1, and the data are expressed as the normalization of the control values.

### 4.6. B16F10 Melanin Content Assay

Melanin content was measured according to methods reported in the literature with appropriate modifications [58]. B16F10 cells were treated according to the experimental conditions for cell redifferentiation. After 24 h, the cells were digested and collected, centrifuged at 2000 rpm for 4 min, the supernatant was removed, the cells were dissolved in 1 mol/L NaOH (10% DMSO) for 250 µL after heating at 80 °C for 1 h and then transferred to a 96-well culture plate, and OD was measured at 470 nm.

Melanin content = Sample OD/Cell counts

### 4.7. Analysis of Cell Apoptosis and Cell Cycle

The FITC annexin V and PI double staining method can detect cell cycle and apoptosis [59]. B16F10 cells were treated with DMEM or drugs for 24 h, and then the cells were collected and washed with PBS. B16F10 cells were stained with FITC An-nexin V and PI for apoptotic evaluation. As regards cell cycle detection, B16F10 cells were stained with PI (BD Biosciences, Franklin Lakes, NJ, USA; cat No. 556547). Fluorescence was measured using a FACSCalibur flow cytometer (FACSCalibur; BD Biosciences, Franklin Lakes, NJ, USA).

### 4.8. RT-PCR and qRT-PCR Assay

The RT-PCR assay detected the mRNA level of cells [60]. RNA was extracted from cells by using a UNIQ−10 Column Trizol Total RNA Isolation Kit (San-gong Co., Shanghai, China) according to the manufacturer’s instructions. According to the manufacturer’s instructions, two micrograms of RNA per sample were converted to cDNA using a PrimeScript1st strand cDNA Synthesis Kit (Takara Bio Inc., Shiga, Japan). The cDNA was used for reverse transcription (RT)-PCR by using *p53*, *p21*, *tyr, tyrp1*, *tyrp2*, *mitf*, *cyclin D1*, *cdk2*, *Bax*, and *bcl*−*2* genes specific primers (Table 1).

### 4.9. Western Blot Analysis

The cells were collected and lysed using RIPA buffer (Solarbio, Shanghai, China) with a protease inhibitor PMSF (Solarbio, China) on ice for 30 min. The supernatant was transferred into new tubes, and protein analyzer Q5000 (Thermo, USA) was used to determine the protein concentration. The denatured protein was used for SDS-PAGE. The following primary antibodies were used: CyclinD1 (Affinity Biosciences, Melbourne, Australia, AF0931), Cdk2 (Affinity Biosciences, AF6237), P53 (Affinity Biosciences, AF0879), P21 (Affinity Biosciences, AF6290), Bcl2 (Boster Biological Technology, Pleasanton, CA, USA, BA0412), Bax (Boster Biological Technology, BA0315−2), β-actin (ZSGB−BIO, Beijing, China).

### 4.10. Statistical Evaluation

The data obtained from different experiments were presented as the mean ± standard deviation (S.D.), calculated from at least three independent experiments and evaluated by ANOVA. Student’s *t*-test for multiple comparisons was used to identify group differences. The values were statistically significant at *p* < 0.05.

## 5. Conclusions

In conclusion, the findings of this study not only contribute to the understanding of chelidonine’s and tetrandrine’s mechanisms of action against melanoma but also illustrate the potential of combining natural compounds as a viable strategy in cancer therapy. This approach aligns with the evolving landscape of cancer treatment, emphasizing targeted, multi-faceted therapies that address the complexity of tumor biology while striving for improved patient outcomes and quality of life. However, the present initial evaluations of potential anti-melanoma therapies from *Chelidonium majus* alkaloids face the true challenge of accurately gauging the efficacy of compounds. It is imperative to emphasize that, despite thorough analyses, additional research is required to ascertain the variability in efficacy across different human melanoma cell lines. 

## Figures and Tables

**Table 1:** Primer sequences of qRT-PCR analyses for mRNA expression.

Gene	Primer Sequence	Annealing Temperature/°C
*p53*	5′-CATCACCTCACTGCATGGAC-3′	58.8
	5′-AAAAGATGACAGGGGCCATG-3′	
*p21*	5′-ATGTCCAATCCTGGTGATGTCC-3′	59
	5′-TCAGGGTTTTCTCTTGCAGAAG-3′	
*cyclin D1*	5′-CTG ACA ACT CTA TCC GCC CC-3′	62
	5′-CAT CCG CCT CTG GCA TTT TG-3′	
*cdk2*	5′-TTCATGGATGCCTCTGCTCTC-3′	60
	5′-TCCAAAAGCTCTGGCTAGTCC-3′	
*bax*	5′-ATGCGTCCACCAAGAAGCTGA-3′	60.95
	5′-AGCAATCATCCTCTGCAGCTCC-3′	
*bcl*−*2*	5′-TTCGCAGACATGTCCAGTCAGCT-3′	61.95
	5′-TGAAGAGTTCCTCCACCACCGT-3′	
*gadph*	5′-CAAGGTCATCCATGACAACTT-3′	59
	5′-GTCCACCACCCTGTTGCTGTA-3′	
*tyr*	5′-CAGGCTCCCATCTTCAGCAGAT-3′	58
	5′-ATCCCTGTGAGTGGACTGGCAA-3′	
*mitf*	5′-GATCGACCTCTACAGCAACCAG-3′	59
	5′-GCTCTTGCTTCAGACTCTGTGG-3′	
*tyrp1*	5′-AGCCACAGGATGTCACTCAGTG-3′	58
	5′-GCAGGGTCATATTTTCCCGTGG-3′	
*tyrp2*	5′-GCAAGATTGCCTGTCTCTCCAG-3′	59
	5′-CTTGAGAGTCCAGTGTTCCGTC-3′	

## Data Availability

The data that support the findings of this study are available from the corresponding author upon reasonable request.

## Supplementary Materials

The following supporting information can be downloaded at: https://www.mdpi.com/article/10.3390/molecules29225412/s1. Figure S1: Heatmap of clustering of the compounds of *Chelidonium majus* L. and *Stephania tetrandra* S. Moore; Figure S2: RNA seq GO enrichment analysis; Figure S3. Chelidonine PCR array results in B16F10 cells; Table S1: Table showing the correlation with melanoma in *Chelidonium majus* L., *Stephania tetrandra* S. Moore, and *Corydalis yanhusuo* herb; Table S2: Table of compounds *Chelidonium majus* L. and *Stephania tetrandra* S. Moore; Table S3: Targets of top50 with degree in PPI; Table S4: Targets of top50 KEGG enrichment analysis list.
