# Supplementary material for: The Synergistic Mechanism of Chelidonium majus Alkaloids on Melanoma Treatment via a Multi-Strategy Insight
Source: Molecules. 2024 Nov 16;29(22):5412. doi: 10.3390/molecules29225412 (PMC11597347; doi:10.3390/molecules29225412)
Supplement: Supplementary file 1 [file molecules-29-05412-s001.zip › molecules-3250524-supplementary.pdf]

Supplementary material

Table S1. Table showing the correlation with melanoma in *Chelidonium majus L.*, *Stephania tetrandra* S. Moore, and *Corydalis yanhusuo* herb

Latin name	Herb database		SymMap database	
	Disease（Melanoma)	P_value	Disease（Melanoma)	P_value
Chelidonium majus	metastatic intraocular melanoma	0.000102539	Stage Ii Acral Lentiginous Melanoma	0.000271319
	Malignant melanoma of conjunctiva	0.000193251		
	Melanoma recurrent	0.001285976		
	Cutaneous Melanoma	1.14E-06		
	melanoma	2.00E-06		
	Melanoma, B16	4.10E-06		
	Melanoma, Experimental	4.55E-06		
	Metastatic melanoma	8.67E-06		
Stephania tetrandra S. Moore	Hereditary Melanoma	0.000128697	Malignant Melanoma Of Vulva	0.000117262
	Lentigo maligna melanoma	0.000131137	Melanoma, Experimental	0.000194924
	Metastatic melanoma	0.000151081	Melanoma, Cutaneous Malignant, Susceptibility To, 1	0.000233367
	Uveal melanoma	0.00017073	Skin Cancer Malignant Melanoma Metastatic	0.000792621
	Stage 0 Cutaneous Melanoma AJCC v6 and v7	0.000672908		
	Advanced Melanoma	0.000801056		
	Stage 0 Skin Melanoma	0.001029366		
	Stage III Cutaneous Melanoma AJCC v6	0.001307472		
	Malignant melanoma of conjunctiva	0.001784392		
	Cutaneous Melanoma	1.20E-08		
	Invasive Cutaneous Melanoma	1.99E-05		
	Melanoma, B16	2.89E-05		
	Superficial spreading malignant melanoma of skin	3.22E-05		
	melanoma	4.00E-05		
	Familial Atypical Mole Melanoma Syndrome	4.27E-05		
	Malignant melanoma, metastatic	8.69E-05		
Corydalis yanhusuo W. T. Wang	Metastatic melanoma	0.00154013	None	
	melanoma	1.26E-05		
	Cutaneous Melanoma	4.19E-05		
	Melanoma, B16	8.61E-07		

Table S2. Table of compounds *Chelidonium majus* L. and *Stephania tetrandra* S. Moore

Pubchem name	smiles
Cryptopine	CN1CCC2=CC(=C(C=C2C(=O)CC3=C(C1)C4=C(C=C3)OCO4)OC)OC
rhoeadine	CN1CCC2=CC3=C(C=C2C4C1C5=C(C(O4)OC)C6=C(C=C5)OCO6)OCO3
Dihydrosanguinarine	CN1CC2=C(C=CC3=C2OCO3)C4=C1C5=CC6=C(C=C5C=C4)OCO6
Protopine	CN1CCC2=CC3=C(C=C2C(=O)CC4=C(C1)C5=C(C=C4)OCO5)OCO3
Dihydrochelirubine	CN1CC2=C3C(=CC(=C2C4=C1C5=CC6=C(C=C5C=C4)OCO6)OC)OCO3
(S)-Canadine	COC1=C(C2=C(CC3C4=CC5=C(C=C4CCN3C2)OCO5)C=C1)OC
chelilutine	C[N+]1=C2C(=C3C(=CC(=C(C3=C1)OC)OC)OC)C=CC4=CC5=C(C=C42)OCO5
(S)-Stylopine	C1CN2CC3=C(CC2C4=CC5=C(C=C41)OCO5)C=CC6=C3OCO6
chelidonine	CN1CC2=C(C=CC3=C2OCO3)C4C1C5=CC6=C(C=C5CC4O)OCO6
Hydroxysanguinarine	CN1C2=C(C=CC3=CC4=C(C=C32)OCO4)C5=C(C1=O)C6=C(C=C5)OCO6
Isocorydine	CN1CCC2=CC(=C(C3=C2C1CC4=C3C(=C(C=C4)OC)O)OC)OC
sanguinarine	C[N+]1=C2C(=C3C=CC4=C(C3=C1)OCO4)C=CC5=CC6=C(C=C52)OCO6
berberine	COC1=C(C2=C[N+]3=C(C=C2C=C1)C4=CC5=C(C=C4CC3)OCO5)OC
Homochelidonine	CN1CC2=C(C=CC(=C2OC)OC)C3C1C4=CC5=C(C=C4CC3O)OCO5
Dihydrochelerythrine	CN1CC2=C(C=CC(=C2OC)OC)C3=C1C4=CC5=C(C=C4C=C3)OCO5
(S)-Scoulerine	COC1=C(C2=C(CC3C4=CC(=C(C=C4CCN3C2)OC)O)C=C1)O
methoxychelidonine	COC1=C2C(=CC3=CC(=C4C5=C(CNC4=C31)C6=C(C=C5)OCO6)O)OCO2
(+)-Chelamidine	CN1CC2=C(C=CC(=C2OC)OC)C3C1C4=CC5=C(C=C4C(C3O)O)OCO5
coptisine	C1C[N+]2=C(C=C3C=CC4=C(C3=C2)OCO4)C5=CC6=C(C=C51)OCO6
2-Propanone,1,3-bis(13,14-dihydro-13-methyl-[1,3]benzodioxolo[5,6-c]-1,3-dioxolo[4,5-i]-phenanthridin-14-yl)-,(R*,S*)-	CN1C(C2=C(C=CC3=C2OCO3)C4=C1C5=CC6=C(C=C5C=C4)OCO6)CC(=O)CC7C8=C(C=CC9=C8OCO9)C1=C(N7C)C2=CC3=C(C=C2C=C1)OCO3
columbamine	COC1=C(C2=C[N+]3=C(C=C2C=C1)C4=CC(=C(C=C4CC3)OC)O)OC
menisperine	C[N+]1(CCC2=CC(=C(C3=C2C1CC4=C3C(=C(C=C4)OC)O)OC)OC)C
Chelerythrine	C[N+]1=C2C(=C3C=CC(=C(C3=C1)OC)OC)C=CC4=CC5=C(C=C42)OCO5
Allocryptopine	CN1CCC2=CC3=C(C=C2C(=O)CC4=C(C1)C(=C(C=C4)OC)OC)OCO3
Corysamine	CC1=C2C=CC3=C(C2=C[N+]4=C1C5=CC6=C(C=C5CC4)OCO6)OCO3
(+)-Chelamine	CN1CC2=C(C=CC3=C2OCO3)C4C1C5=CC6=C(C=C5C(C4O)O)OCO6
N-Methylflindersine	CC1(C=CC2=C(O1)C3=CC=CC=C3N(C2=O)C)C
Berbamine	CN1CCC2=CC(=C3C=C2C1CC4=CC=C(C=C4)OC5=C(C=CC(=C5)CC6C7=C(O3)C(=C(C=C7CCN6C)OC)OC)O)OC
Cycleaneonine	CN1CCC2=CC(=C3C(=C2C1CC4=CC=C(C=C4)OC5=C6C(CC7=CC=C(CO3)C=C7)N(CCC6=CC(=C5OC)OC)C)O)OC
magnoflorine	C[N+]1(CCC2=CC(=C(C3=C2C1CC4=C3C(=C(C=C4)OC)O)O)OC)C
tetrandrine	CN1CCC2=CC(=C3C=C2C1CC4=CC=C(C=C4)OC5=C(C=CC(=C5)CC6C7=C(O3)C(=C(C=C7CCN6C)OC)OC)OC)OC
Isotetrandrine	CN1CCC2=CC(=C3C=C2C1CC4=CC=C(C=C4)OC5=C(C=CC(=C5)CC6C7=C(O3)C(=C(C=C7CCN6C)OC)OC)OC)OC

Pubchem name	smiles
Berbacolorflammine	CN1CCC2=CC(=C3C=C2C1CC4=CC=C(C=C4)OC5=C(C=CC(=C5)CC6=[N+](C=CC7=CC(=C(C(=C67)O3)O)OC)C)OC)OC
(+)-Limacine	CN1CCC2=CC(=C3C=C2C1CC4=CC=C(C=C4)OC5=C(C=CC(=C5)CC6C7=C(O3)C(=C(C=C7CCN6C)OC)O)OC)OC
Curine	CN1CCC2=CC(=C3C=C2C1CC4=CC=C(C=C4)OC5=C6C(CC7=CC(=C(C=C7)O)O3)N(CCC6=CC(=C5O)OC)C)OC
Cissampareine	CN1CCC2=CC(=C(C3=C2C1CC4=CC=C(COC5=C(C=C6CCN=C(C6=C5O)CC7=CC=C(O3)C=C7)OC)C=C4)OC)OC
Cyclanoline	C[N+]12CCC3=CC(=C(C=C3C1CC4=C(C2)C(=C(C=C4)OC)O)O)OC
Hesperetin	COC1=C(C=C(C=C1)C2CC(=O)C3=C(C=C(C=C3O2)O)O)O
Aristololactam	COC1=CC=CC2=C3C4=C(C=C21)NC(=O)C4=CC5=C3OCO5
Javanicin	CC1=C(C(=C2C(=C1O)C(=O)C=C(C2=O)OC)O)CC(=O)C
Hesperidin	CC1C(C(C(C(O1)OCC2C(C(C(C(O2)OC3=CC(=C4C(=O)CC(OC4=C3)C5=CC(=C(C=C5)OC)O)O)O)O)O)O)O)O
Dichotomitin	COC1=CC(=CC(=C1OC)O)C2=COC3=CC4=C(C(=C3C2=O)O)OCO4
Feralolide	CC(=O)C1=C(C=C(C=C1O)O)CC2CC3=C(C(=CC(=C3)O)O)C(=O)O2
Chelidonic acid	C1=C(OC(=CC1=O)C(=O)O)C(=O)O
beta-Isosparteine	C1CCN2CC3CC(C2C1)CN4C3CCCC4
Malathion	CCOC(=O)CC(C(=O)OCC)SP(=S)(OC)OC
citric acid	C(C(=O)O)C(CC(=O)O)(C(=O)O)O
Methylamine	CN
Tyramine	C1=CC(=CC=C1CCN)O
histamine	C1=C(NC=N1)CCN
succinic acid	C(CC(=O)O)C(=O)O
Choline	C[N+](C)(C)CCO
Patchouli alcohol	CC1CCC2(C(C3CCC2(C1C3)C)(C)C)O
Allantoin,(-)-	C1(C(=O)NC(=O)N1)NC(=O)N
Borneol	CC1(C2CCC1(C(C2)O)C)C
136458-42-9	CC1(C2C1C3C(CCC3(C)O)C(=C)CC2)C
CYH	[CH-].[Y]
(-)-Bornyl acetate	CC(=O)OC1CC2CCC1(C2(C)C)C
Z-3-Hexenol	CC/C=C\CCO
l-Menthol	CC1CCC(C(C1)O)C(C)C
9-Oxofarnesol	CC(=CC(=O)CC(=CCCC(=CCO)C)C)C
Aristolene	CC1CCCC2=CCC3C(C12C)C3(C)C
Beta-Gurjunene	CC1CCC2C(C2(C)C)C3C1CCC3=C
(-)-Cis-Carveol	CC1=CCC(CC1O)C(=C)C
Longipinocarvone	CC1(CCCC2(C3C1C2C(=C)C(=O)C3)C)C
beta-Sitosterol	CCC(CCC(C)C1CCC2C1(CCC3C2CC=C4C3(CCC(C4)O)C)C)C(C)C
Homocresol	CCC1=CC(=C(C=C1)O)OC
Tetraneurin A	CC(=O)OCC1CCC2C(C3(C1(CCC3=O)O)C)OC(=O)C2=C
(+)-Camphene	CC1(C2CCC(C2)C1=C)C
Ethanol,2,2,2-triethoxy-	CCOC(CO)(OCC)OCC

**Pubchem name**	**smiles**
Benzaldehyde	C1=CC=C(C=C1)C=O
Guaiene	CC1CCC(=C(C)C)CC2=C1CCC2C
Betaine	C[N+](C)(C)CC(=O)[O-]
Alpha-Muurolene	CC1=CC2C(CC1)C(=CCC2C(C)C)C
delta-amorphene	CC1=CC2C(CCC(=C2CC1)C)C(C)C
Faradiol	CC1C2C3CCC4C5(CCC(C(C5CCC4(C3(CC(C2(CC=C1C)C)O)C)C)(C)C)O)C
2,6,10-Trimethyldodecane	CCC(C)CCCC(C)CCCC(C)C

Table S3. Targets of top50 with degree in PPI

No.	Gene/Protein	Degree	No.	Gene/Protein	Degree	No.	Gene/Protein	Degree
1	PIK3CA	66	18	PIK3CB	35	35	PGR	34
2	CDK2	31	19	PIK3R1	55	36	HSP90AB1	46
3	APP	49	20	PPARG	66	37	ABL1	38
4	LCK	33	21	NFKB1	31	38	SRC	92
5	CTSB	29	22	NR3C1	37	39	ITGB1	42
6	PTPN11	45	23	PTK2B	28	40	ESR1	86
7	ERBB4	31	24	KDR	48	41	IGF1	63
8	FYN	42	25	EGFR	105	42	HIF1A	77
9	MET	30	26	PPARA	50	43	F2	35
10	GSK3B	41	27	KIT	39	44	PLG	36
11	PARP1	36	28	ALB	120	45	MAPK14	52
12	CXCR4	49	29	ACE	34	46	NOS3	43
13	ITGB3	31	30	HSP90AA1	96	47	MDM2	47
14	RXRA	33	31	ESR2	30	48	PDGFRB	34
15	CDK1	33	32	PRKACA	35	49	CREBBP	47
16	AR	51	33	CYP3A4	42	50	CHEK1	30
17	LYN	35	34	REN	31			

Table S4. Targets of top50 KEGG enrichment analysis list

GroupID	Term	Description	LogP
1	hsa05200	Pathways in cancer	-32.01204644
2	hsa05205	Proteoglycans in cancer	-24.26822051
3	hsa05207	Chemical carcinogenesis - receptor activation	-22.12839398
4	hsa04914	Progesterone-mediated oocyte maturation	-16.91642569
5	hsa04014	Ras signaling pathway	-16.11481477
6	hsa05120	Epithelial cell signaling in Helicobacter pylori infection	-10.62245092
7	hsa04659	Th17 cell differentiation	-9.276376314
8	hsa04110	Cell cycle	-8.138245302
9	hsa04148	Efferocytosis	-6.651391767
10	hsa04540	Gap junction	-6.452931287
11	hsa04924	Renin secretion	-5.280304731
12	hsa05410	Hypertrophic cardiomyopathy	-4.692142492
13	hsa04916	Melanogenesis	-4.622961357
14	hsa04064	NF-kappa B signaling pathway	-4.572932065
15	hsa04976	Bile secretion	-3.361730119

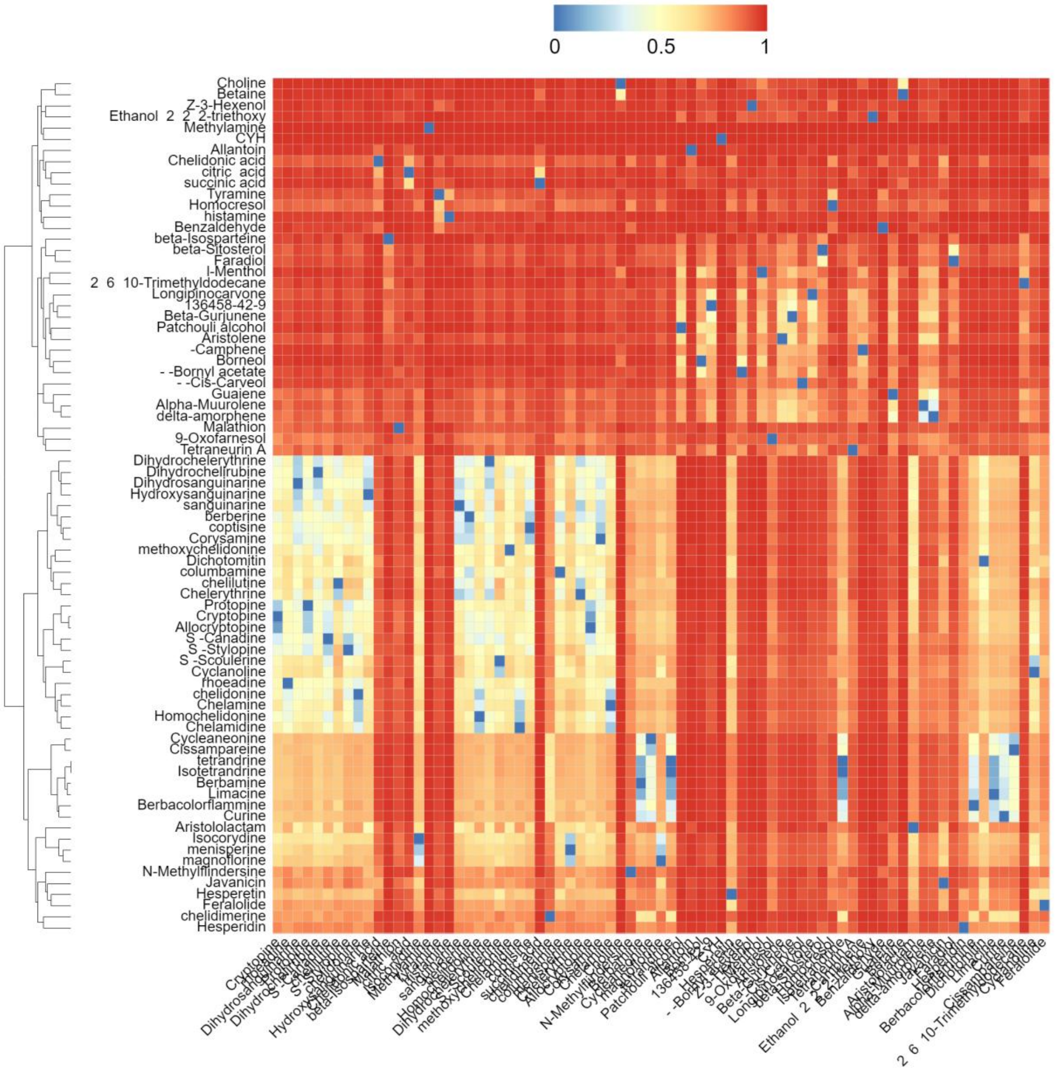

**Options**	Physicochemical Properties Heatmap: None, Heatmap: distance matrix, Properties Color and Display Values: Z-scores, Linkage Method: single

Figure S1. Heatmap of clustering of the compounds of *Chelidonium majus* L. and *Stephania tetrandra* S. Moore

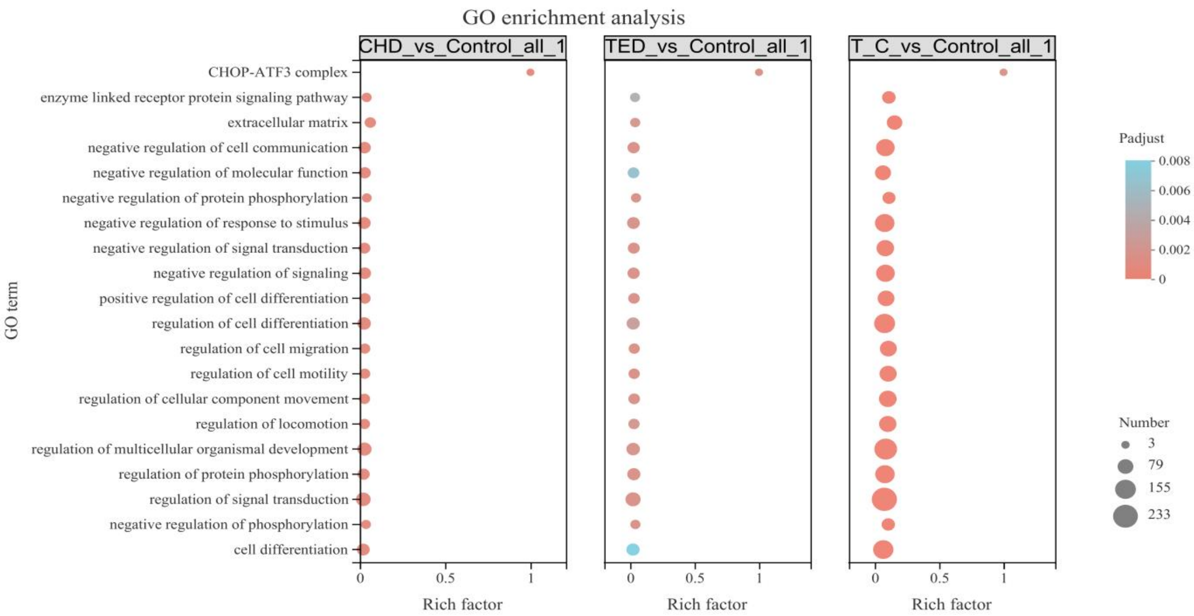

Figure S2. RNA seq GO enrichment analysis

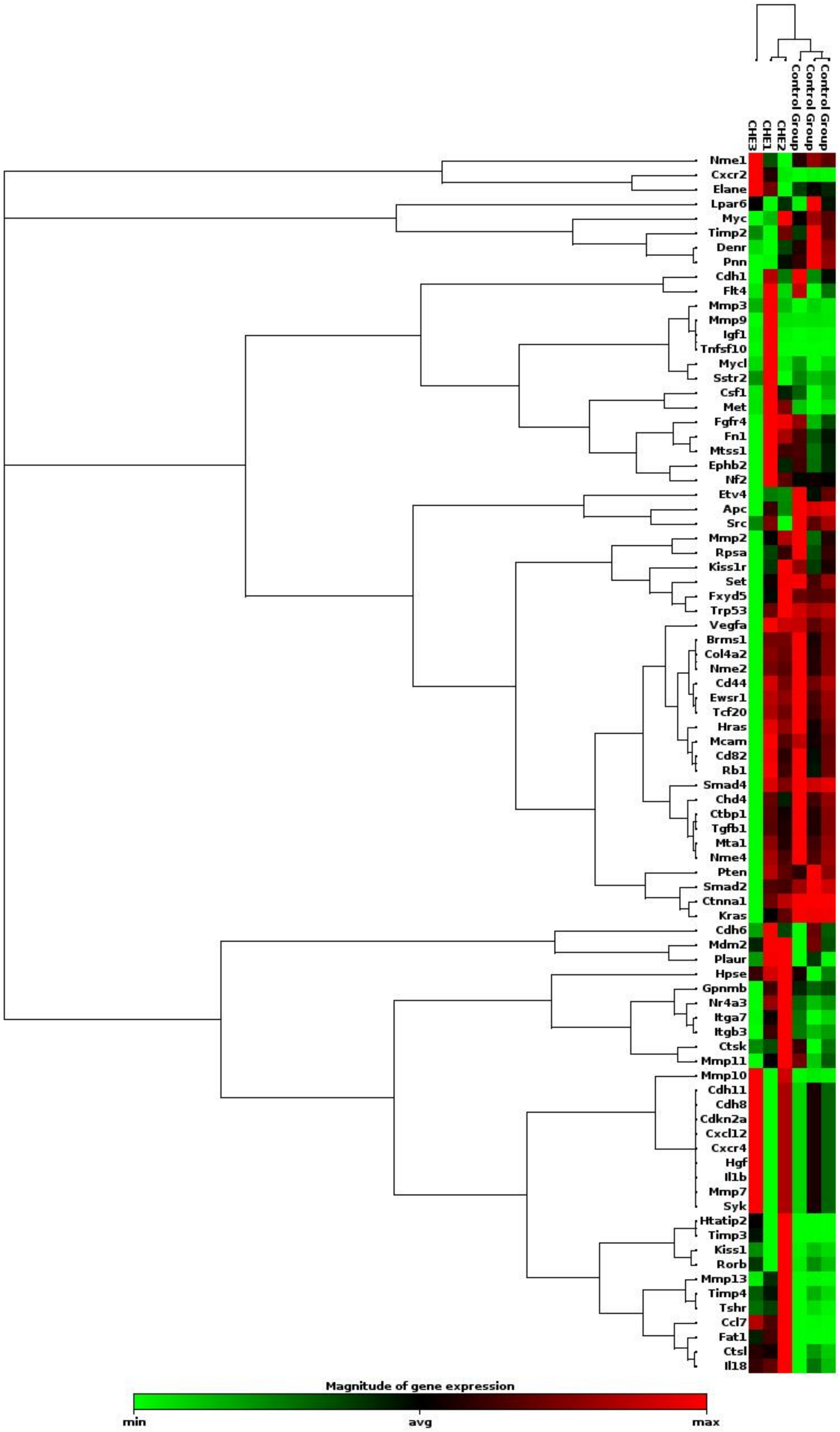

Figure S3. Chelidonine PCR array results in B16F10 cells
